# Clinical Profile and the Extent of Disability in Multiple Sclerosis Patients in Madinah, Saudi Arabia

**DOI:** 10.7759/cureus.25851

**Published:** 2022-06-11

**Authors:** Esraa Q Alsaedi, Marwa Q Alsaedi, Farah A Mansuri

**Affiliations:** 1 Preventive Medicine, Saudi Board of Preventive Medicine, Ministry of Health, Madinah, SAU; 2 Family Medicine, Ministry of Health, Madinah, SAU; 3 Family and Community Medicine, Taibah University, Madinah, SAU

**Keywords:** edss, clinical profile, ms disability, madinah, multiple sclerosis

## Abstract

Objectives: The objective is to study the demographics and clinical characteristics of Saudi multiple sclerosis (MS) patients in Madinah, Saudi Arabia, and assess their extent of disability using the Expanded Disability Status Scale (EDSS).

Methods: This hospital-based study intended to address the population of all MS-diagnosed patients registered between 2018 and 2021 in the Neurology Department of King Fahad Hospital in the Madinah region. Data were gathered from medical records and by interviewing participants in the Neurology Clinic. The chi-square test and linear and logistic regression were applied to draw inferences.

Results: A total of 195 MS-diagnosed patients were included in the analysis. Of these, 72.3% were female. The mean age of the total sample was 34.9±9.2 years, and 7.7% reported a positive family history. Of all patients, 17.9% (n=35) had comorbidities. The mean age at diagnosis was 29.3±8.2 years. The majority (85.6%) were diagnosed with relapsing-remitting multiple sclerosis (RRMS); 77.9% had an EDSS score between 0.0 and 1.5, showing little or no disability. A statistical significance existed between EDSS and the current age of the patients (p=0.004), age at onset (p=0.007), type of MS (p=0.000), presence of muscle weakness (p=0.044), bladder or bowel difficulties at onset (p=0.043), and the duration of MS (p=0.000). Of the patients, 23.6% were not using disease-modifying therapy (DMT). The most commonly used drug was interferon beta 1-b.

Conclusion: A lower EDSS was associated with younger age, diagnosis at a younger age, RRMS, duration less than five years, and lower body mass index (BMI). To increase the generalizability of findings, a national MS registry system and further prospective analytical epidemiological research studies are recommended.

## Introduction

Multiple sclerosis (MS) is a chronic inflammatory disease involving the central nervous system (CNS). It is considered an autoimmune demyelinating disease that damages CNS neurons [1]. The most commonly affected sites are the brain stem, spinal cord, optic nerve, and periventricular white matter [2].

The disease onset often affects young adults aged between 20 and 40 years, and it can less often appear during childhood, adolescence, and late adulthood [3]. Almost 4%-10% of patients diagnosed with MS are under 16 years of age [4]. Globally, MS affects females twice more than males, and some individuals are reported to have a genetic disposition. Some countries have reported a higher female-to-male ratio of 4:1 [5].

The prevalence of MS as defined by the Kurtzke classification is low at <5/100,000, medium at 5-29/100,000, and high at ≥30/100,000 [6]. The MS prevalence increased worldwide in 2020 to 2.8 million, 30% higher than the MS Atlas 2013 estimation [5].

In Middle Eastern countries, the prevalence is low to moderate according to the MS Atlas 2013, but recent studies have reported a moderate to high prevalence, and it has been increasing in the last few years [6-8]. In Saudi Arabia, data are lacking regarding disability among MS patients and the epidemiological profile. However, the most recent data estimated a high and increasing incidence in the country of 40.40/100,000 [9].

The symptoms of MS are spasticity, fatigue, movement disorders, sexual dysfunction, bladder and bowel dysfunction, depression, pain, speech disorders, cognitive difficulties, and dysphagia. The severity and progression of the symptoms are variable from one person to another, with the expression of manifestation varying over time for the same person [10]. Neurological symptoms, the clinical course of the disease, and the severity of symptoms are measured by a scale designed for MS patients known as the Expanded Disability Status Scale (EDSS) [11].

The demographics and clinical characteristics of MS are well-documented in Japanese and Caucasian populations [12,13]. However, in the Middle East and Saudi Arabia, data have been limited regarding the disease clinical features, demographics, and disability of MS in Madinah [14]. A recent study conducted in the southwest region of Saudi Arabia described the demographics and disease characteristics in that region. It reported that optic symptoms at onset were presented in 37.8% of their 82 MS patients [15]. A case-control study in the western region investigated the risk factors for MS development and reported a brief insight into the demographics, mainly on the age of the patient and the age at onset; 35% were male and 65% were female out of 80 MS cases [16].

This study was conducted to identify the demographics and clinical profile of Saudi MS patients in Madinah, Saudi Arabia, and assess the extent of their disability using the EDSS.

## Materials and methods

This hospital-based study intended to address the population of all MS diagnosed patients registered between 2018 and 2021 in the Neurology Department of King Fahad Hospital in the Madinah region. Data were gathered from medical records and by interviewing participants in the Neurology Clinic on their scheduled day of attendance or by phone to gather any missing data that the medical records could not provide.

Patients were included in this study if they were aged 16 years or older; regularly attended their follow-up visits; and were diagnosed with MS or clinically isolated syndrome (CIS), based on the 2017 revised McDonald criteria, and registered under the diagnosis code G35.0, which refers to MS as per the International Classification of Diseases, Tenth Revision (ICD-10). Non-Saudis, patients with neuromyelitis optica or isolated transverse myelitis, and patients with incomplete medical data or unconfirmed MS diagnosis were excluded.

The data collection procedure was conducted in the Neurology Department at King Fahad Hospital. This is the main referral neurology center for MS cases in the Madinah region. Ethical approval was obtained from the Institutional Review Board (IRB), General Directorate of Health Affairs in Madinah (approval number: H-03-M-084). Data collection included each patient’s age, sex, marital status, date of onset and diagnosis, type of MS, duration of disease, family history of MS or autoimmune diseases, comorbidities, EDSS score, number of relapses, and current patient treatment.

Descriptive and inferential data were analyzed using IBM SPSS Statistics for Windows, version 22.0 (IBM Corp., Armonk, NY, USA). Continuous data were summarized with means ± SD and ranges. Spearman correlation coefficients were calculated to evaluate the associations between variables. Categorical data were reported as percentages and frequencies; a chi-square test was also used. Linear regression and multivariate logistic regression analyses were used to obtain the appropriate model. A p-value less than 0.05 was considered statically significant.

## Results

A total of 195 MS-diagnosed patients were included in the study. The sex distribution showed 72.3% (n=141) of the study sample were female, while male patients represented 27.7% (n=54), with a 2.6:1 female-to-male ratio. Of the patients, 44.1% were from 26 to 35 years of age, with a mean age of 34.897±9.185 years; 51.8% (n=101) were married, and 51.3% (n=100) were employed. A total of 9.2% were obese, and most of the cases had neither comorbidity nor family history (82.1%, n=160; 92.3%, n=180, respectively). Only 7.7% reported positive family history, of whom 7.2% of patients were diagnosed with RRMS and 0.5% had secondary progressive MS (SPMS), with the vast majority women (5.1%). The data showed that 17.9% of the patients had comorbidities, with 37.1% diabetic, 31.4% hypertensive, 20% with thyroid disease, and 17.1% and depression (Table 1).

**Table 1:** MS patients’ demographic characteristics (n=195). BMI: body mass index

Demographic data		n	%
Sex	Male	54	27.7
	Female	141	72.3
Age Group	25 and younger	27	13.8
	26–35	86	44.1
	36–45	61	31.3
	46–55	16	8.2
	56 and older	5	2.6
Marital Status	Single	82	42.1
	Married	101	51.8
	Divorced	8	4.1
	Widowed	4	2.1
Employment Status	Employed	100	51.3
	Unemployed	95	48.7
BMI Category	Underweight	3	1.5
	Normal weight	120	61.5
	Overweight	54	27.7
	Obese	18	9.2
Comorbidities	No comorbidity	160	82.1
	Yes	35	17.9
Family History	No family history	180	92.3
	Yes	15	7.7

**Figure 1:**
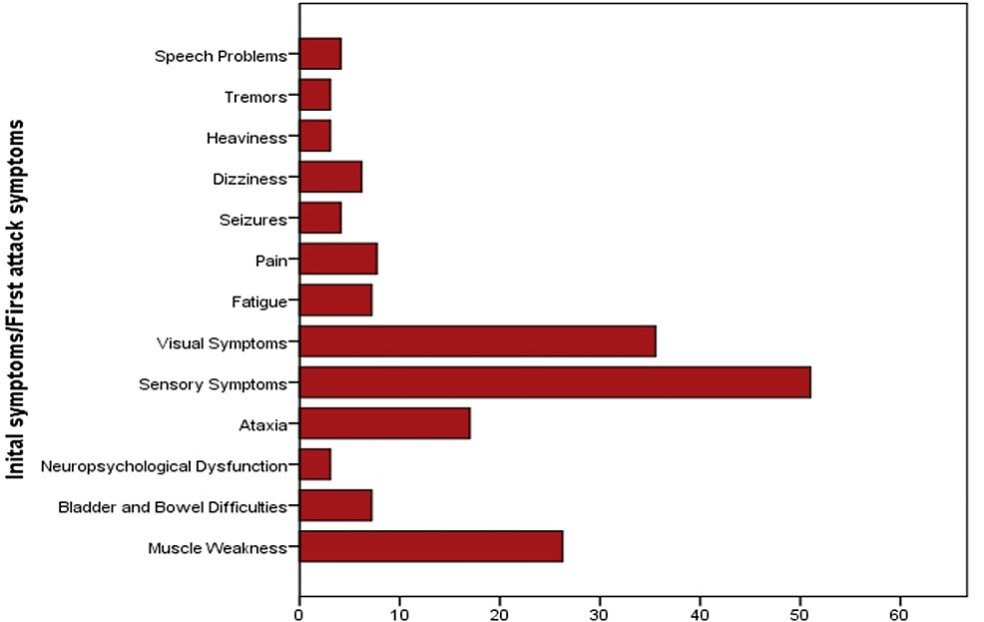
Most common presentation at onset

Table 2 describes the clinical characteristics of the cases. The age at diagnosis ranged between 15 and 52 years old, with a mean age of 29.3±8.2 years. More than half (60.5%) of the cases reported their initial attack symptoms before age of 30 years. No significant difference existed by sex in the age at diagnosis (p=0.297). The majority of the cases were diagnosed with RRMS type (85.6%). Patients with RRMS developed their initial symptoms at an earlier age compared to those with other types of MS (p=0.045). Sensory symptoms were the most frequent presentation in 51% of the patients, then visual symptoms in 35.6%, followed by muscle weakness in 26.3% (Figure 1). The mean MS duration was 5.64±4.45 years. The mean number of relapses was 1.06±1.4 in two years.

**Table 2:** The clinical profile of the cases. MS: multiple sclerosis; RRMS: relapsing-remitting MS; PPMS: primary progressive MS; SPMS: secondary progressive MS ;CIS: clinically isolated syndrome; EDSS: expanded disability status scale

Clinical profile data		n	%
Age Group	25 and younger	70	35.9
	26–35	81	41.5
	36–45	37	19.0
	46–55	7	3.6
Type of MS	RRMS	167	85.6
	PPMS	12	6.2
	SPMS	10	5.1
	CIS	6	3.1
EDSS Score	0.0–1.5	152	77.9
	2.0–3.5	20	10.3
	4.0–5.5	15	7.7
	≥6	8	4.1
MS Duration	Up to 2 years	59	30.3
	3–10 years	112	57.4
	11–27 years	24	12.3
Number of Relapses	No relapses	77	39.5
	1–2 relapses	95	48.7
	More than 2 relapses	23	11.8

 

**Figure 2:**
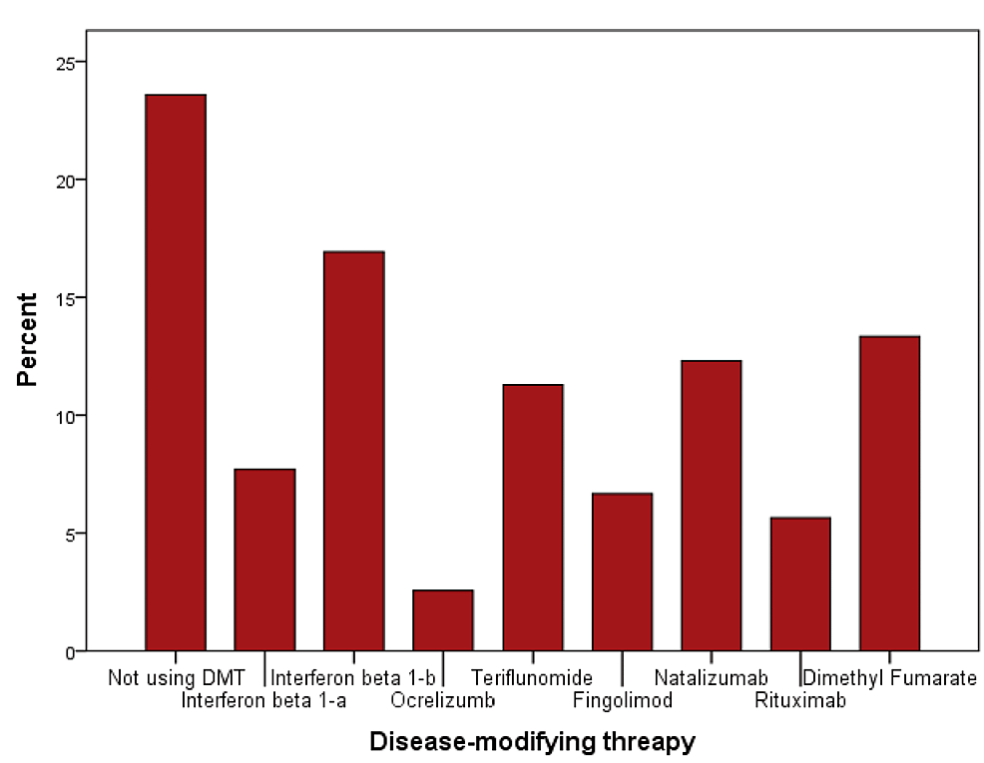
Most commonly used disease-modifying drugs

Our sample showed little or no disability among 77.9% (n=152) of patients, with EDSS scores ranging from 0.0 to 1.5. Only 4.1% (n=8) had a severe disability, with an EDSS score of more than 6. A statistical significance using the chi-square test existed between the EDSS score and the current age of the patients (p=0.004), age at onset (p=0.007), type of MS (p=0.000), the presence of muscle weakness (p=0.044) and bladder or bowel difficulties (p=0.043) at onset, and duration of MS (p=0.000). Out of the total 195 patients, 23.6% were not on disease-modifying therapy (DMT). The most commonly used DMT was interferon beta 1-b (Figure 2).

It was estimated that lower EDSS scores were significantly associated with OR= 3.7 (95% CI 1.4-9.3), 3.3 (95% CI 1.3-8.2), and 2.6 (95% CI 1.04-6.5) among younger patients, those diagnosed at an early age and BMI normal to overweight, respectively. Similarly, lower EDSS scores were significantly associated with RRMS and shorter duration of illness of less than five years with OR=6.5 (95% CI: 2.5-17.1), and 2.9 (95% CI: 1.1-7.3), respectively.

Multiple linear regression was done to predict EDSS scores from patients’ current age, age at diagnosis, BMI, duration, type of MS, and presence of hypertension. The model predicted EDSS scores with statistical significance: F (3, 191)=11.486, p˂0.000, R2=0.153. BMI, type and duration of MS were statically significant to the prediction (p<0.05; Tables 3, 4 ).

**Table 3:** Regression model ^a ^ANOVA applied; dependent variable: EDSS mean value ^b ^predictors: (constant), duration of MS, BMI, type of MS df: degrees of freedom

Model	Sum of Squares	df	Mean Square	F	P-value
Regression	92.684	3	30.895	11.486^a^	.000^b^
Residual	513.734	191	2.690		
Total	606.418	194			

**Table 4:** Estimated model coefficients: multiple linear regression B: unstandardized coefficients; SE: standard error; Beta: standardized coefficients; CI: confidence interval; VIF: variance inflation factor

Model	B	SE	Beta	t	P-value	95% CI		Collinearity Statistics	
						Lower Bound	Upper Bound	Tolerance	VIF
(Constant)	-2.908	.986		-2.949	.004	-4.853	-.963		
BMI	.083	.035	.159	2.385	.018	.014	.152	.993	1.007
Type of MS	.608	.172	.239	3.532	.001	.269	.948	.973	1.028
Duration of MS	.683	.190	.243	3.587	.000	.308	1.059	.966	1.035

Logistic regression was performed to determine the effects of age, BMI, number of relapses, DMT, employment status, marital status, comorbidities, type and duration of MS on the likelihood of patients having a higher EDSS score. The logistic regression model was statistically significant: χ2(13)=47.031, p<0.0005. The model explained 41.5% of the variance in EDSS scores and correctly classified 92.3% of cases. Those diagnosed with other types of MS were 4.5 (95% CI 1.16-17.637) times more likely to exhibit higher EDSS scores than those with RRMS. Increasing age was associated with an 11.3% increased likelihood (95% CI 1.035-1.197) of exhibiting a higher EDSS score. Increasing duration of MS was associated with a 14.3% increase in the likelihood (95% CI 1.0-1.3) of having higher a EDSS score. An increase in one unit of BMI was associated with a 14.9% (95% CI 1.01-1.3) increase in odds of having a higher EDSS score (Table 5).

**Table 5:** Predictors of higher EDSS score ^a ^reference category\ ^b^ includes divorced and widowed ^c ^significant P-value RRMS: relapsing-remitting multiple sclerosis; B: constant coefficient; SE: standard error; Exp(B): odds ratio; CI: confidence interval

Variables		B	SE	P-value	Exp(B)	95% CI	
						Lower	Upper
Age		.107	.037	.004 ^c^	1.113	1.035	1.197
Sex	Female ^a^	-.656	.719	.361	.519	.127	2.122
	Male						
Employment status	Employed	-.246	.572	.668	.782	.255	2.400
	Unemployed ^a^						
BMI		.139	.067	.040 ^c^	1.149	1.007	1.311
Duration of MS		.134	.068	.049 ^c^	1.143	1.001	1.306
Comorbidities	Yes ^a^	.220	.704	.755	1.246	.314	4.949
	No						
Number of relapses		.249	.176	.156	1.283	.909	1.812
Type of MS	RRMS ^a^	1.512	.693	.029 ^c^	4.535	1.166	17.637
	Other types						
Marital status	Single ^a^			.461			
	Married	-.844	.753	.262	.430	.098	1.879
	Others ^b^	.059	1.052	.955	1.061	.135	8.340
DMT	Yes ^a^	-.779	.800	.330	.459	.096	2.200
	No						
Cholecalciferol vit D3	Yes^ a^	-.156	.671	.816	.856	.230	3.190
	No						
Vit B complex	Yes ^a^	.014	.681	.984	1.014	.267	3.849
	No						

## Discussion

MS is a disabling, progressive CNS inflammatory disease, mainly affecting young adults who are in the most productive periods of their lives. MS is rapidly progressive and changing in many regions of the world. It is considered one of the non-traumatic causes of disability in the long term. Therefore, it impacts the healthcare costs, loss of productivity, and quality of life of patients [5,14].

Saudi Arabia does not have a long history of research in MS. This study described the demographics and clinical characteristics of 195 MS patients. We found that 44.1% of patients were from 26 to 35 years of age, with a mean age of 34.897±9.185 years. This was similar to a study that attempted to create an MS registry from 2015 to 2018 across the Kingdom, including 20 hospitals and 2,516 patients, where the median age of the population was 32 years [9]. Halawani et al. found a median age of 32 years in the western region of Saudi Arabia. Al-Abdullah et al. had similar results, with a mean age of 34.57±9.48 years in the southwest cities of Saudi Arabia. In Kuwait, Alroughani et al. found the mean age of their cohort was 33.7 years [15-17].

The mean age at diagnosis was 29.27±8.18 years in our study, ranging between 15 and 52 years, and more than half (60.5%) of the cases reported their initial attack symptoms before 30 years of age. This could be due to the advances in recent years in the diagnosis and increasing awareness among medical staff and the community helping to catch the disease at a younger age. These results are consistent with Halawani et al. and a published meta-analysis of 52 studies in the Middle East and North Africa (MENA) countries that found the overall estimated mean age at diagnosis was 28.54 years (27.61-29.48 years) [16,18]. Surprisingly, we observed that patients with RRMS developed their initial symptoms at an earlier age compared to those with other types of MS (p=0.045). This could be related to the age of our patients since most of them were younger. However, Al-Abdullah et al. found male patients had an onset at a significantly younger age compared to female patients (p=0.003) [15].

Globally, the MS female-to-male ratio is high; our ratio was 2.61:1, as in Dubai-native MS patients in Inshasi et al. [19]. Furthermore, since 2013, the ratio in some countries has doubled to 4:1. However, it was slightly lower at 1.33:1 in Qatari patients in Deleu et al. [5,8]. Among our cases, a positive family history existed for 7.7%. This is in line with Halawani et al. in the western region of Saudi Arabia at 6.2%, Deleu et al. in Qatar at 10.4%, and Inshasi et al. in Dubai at 10.6. In contrast, AlJumah et al. in Saudi Arabia found 12.8%, and Alroughani in Kuwait found 13.32%, reporting a higher positive family history among their study individuals [8,16,19-21]. However, some studies found lower rates, such as Hamdy et al. in Egypt at 2.28% and Al-Abdullah et al. in the southwestern region of Saudi Arabia at 4.8% [15,22].

In this study, the most observed MS presentation at onset was sensory symptoms (51%), then visual symptoms (35.6%), followed by muscle weakness (26.3%). Similar results were also reported by a prospective and retrospective hospital-based study conducted at Hamad General Hospital in Qatar in April 2010. It found that sensory manifestations were the most common symptoms. In Kuwait, Alroughani et al. also observed that 49.2% of patients had sensory, 29.7% had a motor, and 27.3% had visual manifestations [8,17]. This is similar to an Iranian study that observed that 51.7% and 47.5% of the initial symptoms of their participants were sensory and visual symptoms [23].

In comparison to other regional studies, a recent retrospective study was conducted to describe the demographics and disease characteristics of 82 MS cases in the Aseer Central Hospital, Abha, and the Armed Forces Hospital, Khamis Mushayt. It reported that optic symptoms at onset presented in most (37.8%) of their study cases [15]. However, Heydarpour et al. conducted a systematic review and meta-analysis regarding MS epidemiology and identified that weakness was the predominant symptom for MS patients in Saudi Arabia [18]. These results concur with the findings of a cross-sectional study in Saudi Arabia by AlJumah et al. The researchers found that muscle weakness was the most common symptom at the first attack (57.1% of 2,516 MS cases), followed by visual manifestations (48.2%) [9]. Similarly, Inshasi et al. performed a hospital-based retrospective study in the UAE, and motor symptoms were the commonest (72.78% of 284 MS cases) [19]. The differences in these findings could be explained by several factors. First, different patient assessment methods were used, along with different environmental and genetic factors related to the region. Second, patients tend to seek consultations based on the severity of their symptoms, which depends on the threshold each can bear. Lastly, the ascertainment of data during the patient’s initial attack is difficult because it could present multiple foci affecting more than one neural axis pathway.

The majority of our patients (77.9%) had little to no disability, with EDSS scores ranging from 0.0 to 1.5, and only 4.1% had a severe disability with an EDSS score of more than 6. This is comparable to the findings of AlJumah et al. [9]. This present study assessed the clinical and demographic features of MS and their relation to EDSS severity. We found a correlation between the EDSS score and the current age of the patients (p=0.004). Younger patients under 35 years old scored an average of 3.671 (95% CI OR 1.434-9396) and were likely to have a lower EDSS score. Liguori et al. found that the EDSS score increased with age (p<0.0001) [24]. Similarly, Xue et al. reported that RRMS patients in that age group with EDSS scores ≤3 had statistically significant differences from those with an EDSS score of >3 (p<0.05) [25].

Our results showed a significant association between EDSS score and the age of our patients at onset (p=0.007). Trojano et al. and Liguori et al. observed similar findings in their cohorts [24,26]. In contrast, Minden et al. measured disability using the disease step method and recorded that age at diagnosis was significantly correlated with disability (p<0.001) [27]. The subtype of MS was correlated with disability (p=0.000). This agrees with Minden et al. findings [27]. Many studies have concluded a relationship between the MS duration and EDSS score. This was consistent with our study finding that patients with a duration of less than five years were 2.938 times (95% CI OR 1.181-7.308) more likely to have a lower EDSS score [21,24,28].

Patients with lower BMI were 2.6 times more (95% CI 1.04-6.5) likely to have a lower EDSS score, and every increase of one unit of BMI had a 14.9% (95% CI 1.01-1.3) increase in the odds of having a higher EDSS score. Ben-Zacharia et al. similarly concluded that the odds for EDSS to increase at least one point in obese individuals were eight times higher than in patients with a normal BMI [29]. Zamzam et al. also showed that higher-BMI patients had higher EDSS scores (95% CI 0.17-0.21). These findings were contrary to those of Tadić et al., who found no evidence of a significant association between BMI and EDSS score [28,30].

One of the limitations of this study is that it was conducted in one major hospital in the Medinah region and thus cannot be generalized to all MS patients of Saudi Arabia. However, this study documented the demographics and clinical characteristics in Madinah and investigated their relationship with the disability of the patients. Another limitation may be that the number of MS patients was underestimated since some of the patients, such as those who lived in the provinces of Madinah, were unable to attend the regional treatment hospital.

## Conclusions

This study provided a baseline of demographic and clinical characteristic data of MS patients in the Medinah region. Lower EDSS scores were significantly associated with younger age, diagnosis at an early age, RRMS type, duration less than five years, and lower BMI.

A national MS registry will be useful to help understand the natural history of the disease, improve the follow-up process of patients, avoid the duplication of files of the same patient across the country, and aid in promoting a good-quality epidemiological research study. Large prospective epidemiological research studies in the future are recommended. High-quality and local epidemiological data can promote better insight to improve the understanding of MS risk and may reduce the costs of the health system because it mainly affects young adults.
